# Increased Cell Surface Free Thiols Identify Effector CD8^+^ T Cells Undergoing T Cell Receptor Stimulation

**DOI:** 10.1371/journal.pone.0081134

**Published:** 2013-11-13

**Authors:** Samuel Troy Pellom, Ryan D. Michalek, Katie E. Crump, P. Kent Langston, Daniel G. Juneau, Jason M. Grayson

**Affiliations:** 1 Laboratory of Lymphocyte Function, Meharry Medical College, Nashville, Tennessee, United States of America; 2 Metabolon Corporation, Durham, North Carolina, United States of America; 3 Department of Microbiology and Immunology, Wake Forest University School of Medicine, Winston-Salem, North Carolina, United States of America; University of Iowa, United States of America

## Abstract

Recognition of peptide Major Histocompatibility Complexes (MHC) by the T cell receptor causes rapid production of reactive oxygen intermediates (ROI) in naïve CD8^+^ T cells. Because ROI such as H_2_O_2_ are membrane permeable, mechanisms must exist to prevent overoxidation of surface proteins. In this study we used fluorescently labeled conjugates of maleimide to measure the level of cell surface free thiols (CSFT) during the development, activation and differentiation of CD8^+^ T cells. We found that during development CSFT were higher on CD8 SP compared to CD4 SP or CD4CD8 DP T cells. After activation CSFT became elevated prior to division but once proliferation started levels continued to rise. During acute viral infection CSFT levels were elevated on antigen-specific effector cells compared to memory cells. Additionally, the CSFT level was always higher on antigen-specific CD8^+^ T cells in lymphoid compared to nonlymphoid organs. During chronic viral infection, CSFT levels were elevated for extended periods on antigen-specific effector CD8^+^ T cells. Finally, CSFT levels on effector CD8^+^ T cells, regardless of infection, identified cells undergoing TCR stimulation. Taken together these data suggest that CD8^+^ T cells upregulate CSFT following receptor ligation and ROI production during infection to prevent overoxidation of surface proteins.

## Introduction

 CD8^+^ T cells are critical for protection from intracellular pathogens such as viruses and certain bacteria and are critical to tumor control. Prior to infection naïve CD8^+^ T cells circulate through the spleen and secondary lymphoid organs surveying professional antigen presenting cells for their cognate antigen. In the absence of cognate stimulation they die within six months [1]. But if antigen is encountered along with costimulation and inflammatory cytokines, naïve CD8^+^ T cells differentiate into effector cells. As part of this programmed differentiation CD8^+^ T cells now express molecules such as perforin and granzymes that are essential to killing infected cells and cytokines such as interferon gamma (IFNγ), tumor necrosis factor alpha (TNFα) and interleukin-2 (IL-2). In addition to expression of cytolytic molecules, effector cells undergo 10 to 12 divisions to amplify numbers with expansion peaking ~ one week later. From days 8 to 35 the number of antigen-specific cells declines 10 to 20-fold. The surviving antigen-specific CD8^+^ T cells differentiate into memory CD8^+^ T cells that undergo a slow homeostatic proliferation to maintain numbers and are able to rapidly respond during secondary infection to control disease [2]. Understanding the mechanisms that control CD8^+^ T cell activation, proliferation and differentiation is critical not only for vaccine development but also for graft rejection and cancer therapy.

Prior work from our laboratory has demonstrated that activation of naïve CD8^+^ T cells elicits ROI production [3]. This increase is essential because antioxidants that lower ROI, decrease activation and proliferation [4]. The mechanisms by which ROI control T cell activation have been the subject of intense research. Since ROI can oxidize all macromolecules determining the relevant oxidation targets is essential. Reversible modulation of cysteine oxidation is an attractive mechanism for ROI to influence cellular function. Indeed studies by our laboratory and others have demonstrated that activation increases sulfenic acid (-SOH), the first oxidation product of cysteine, both in the total proteome, and in protein tyrosine phosphatases such as SHP-1 and SHP-2 [3,5]. The reversible formation of this molecule is absolutely necessary for the proliferation of CD4^+^ and CD8^+^ T [3] and B cells [6]. To date most studies have focused on intracellular redox events, but evidence suggests cysteine oxidation at the cell surface is essential for T cell activation and function. In 1980 Redelman et. al [7] demonstrated that allogeneic cell-mediated lysis required surface sulfhydryl groups as thioylte monoquat, which is not cell permeant, decreased killing. Smith and colleagues demonstrated that free thiol binding reagents including N-ethylmaleimide (NEM) were able to inhibit T cell proliferation through the loss of IL-2 responsiveness [8]. These findings were extended by Lawrence et al. who demonstrated that 5,5’-dithiobis-(2-nitrobenzoic acid) (DTNB), a cell impermeant surface thiol binding reagent, was able to inhibit proliferation of human PBMCs [9]. Taken together they suggest that redox regulation of CSFT is critical for T cell activation and function. How CSFT are regulated during the development and differentiation of antigen-specific CD8^+^ T cells during a physiological response such as viral infection is unknown.

In this study we used fluorescently labeled conjugates of maleimide and MHC Class I tetramers to examine how CSFT levels were modulated during CD8^+^ T cell development, activation, and differentiation following viral infection. We found that developing CD8 SP T cells had increased CSFT relative to CD4 SP or CD4CD8 DP T cells in the thymus. Once CD8^+^ T cells emigrated to the periphery CSFT levels were higher on activated/memory compared to naïve phenotype CD8^+^ T cells. After activation CSFT levels increased prior to division and became further elevated as cells proliferated. During acute viral infection CSFT levels were elevated on antigen-specific effector cells compared to memory cells. Additionally, the CSFT level was always higher in lymphoid compared to nonlymphoid organs. During chronic viral infection, CSFT levels were elevated for extended periods on antigen-specific effector CD8^+^ T cells. Finally, CSFT levels on effector CD8^+^ T cells, regardless of infection, identify cells undergoing recent TCR stimulation, not cytolytic capacity or proliferative status. Taken together these data suggest that CD8^+^ T cells upregulate CSFT following receptor ligation and ROI production to prevent overoxidation of surface proteins.

## Materials and Methods

### Mice, virus and infections

Six to 8-week old female C57BL/6 mice were purchased from the National Cancer Institute (Fredericksburg, MD). Mice were infected with 2x10^5^ p.f.u. of LCMV-Armstrong i.p., or 2x10^6^ p.f.u. of LCMV-Clone 13 i.v. and used at the indicated timepoints. For secondary infection mice were rechallenged greater than 60 days postinfection following LCMV-Armstrong. All studies were approved by the Institutional Animal Care and Use Committee (IACUC) of the Wake Forest University School of Medicine (Animal welfare assurance number A3391-01). Virus stocks were grown and quantitated, as described previously [10].

### Priming of transgenic cells

Priming of P14 transgenic cells has been described before [11]. In these studies either 10^4^ or 10^5^ naïve P14 Thy1.1^+^ cells were transferred into naïve C57BL/6 (Thy1.2^+^) mice, challenged with LCMV-Armstrong, and used at the indicated timepoints.

### Cell isolation

The spleen was removed from mice after cervical dislocation. Following mechanical disruption of splenocytes on a wire mesh screen, red blood cells were removed by osmotic lysis in ACK lysis buffer (Lonza). Splenocytes were then resuspended in RPMI 1640 supplemented with 10% FCS (Hyclone), L-glutamine (Hyclone), penicillin-streptomycin (Cellgro), and β-mercaptoethanol (Gibco). Bone marrow cells were extracted from both femurs using a 23 gauge syringe and lysed in ACK buffer to remove red blood cells. Lymph node cells were isolated following mechanical disruption on a wire mesh screen. Peripheral blood mononuclear cells (PBMC) were isolated following retroorbital bleeding by Histopaque (Sigma) gradient centrifugation. For isolation of nonlymphoid tissues, mice were euthanized, the abdomen was opened, the hepatic vein was cut, and 5 ml of ice-cold PBS was injected directly into the hepatic artery to perfuse the liver. The gall bladder was removed, and the entire liver was excised. The liver tissue was homogenized with a wire screen and incubated in 0.25 mg of collagenase B/ml (Boehringer Mannheim) and 1 U of DNase (Sigma /ml at 37°C for 45 min. Digested liver was centrifuged, and the pellet was resuspended in 5 to 10 ml of 44% Percoll (Sigma). This solution was underlaid with 56% Percoll and spun at 2,000 rpm for 20 min at 20°C. The intrahepatic lymphocyte populations were harvested from the interface, and red blood cells were lysed with 0.83% ammonium chloride, washed, and counted. Lung lymphocytes were isolated in a similar manner.

### CD8^+^ T cell purification

CD8^+^ T cells were negatively selected by magnetic bead enrichment from the spleens of naïve 6-8 week old mice using the Miltenyi MicroBead system according to the manufacturer’s protocol. Purity was >95% as determined by flow cytometry.

### CFSE labeling

CFSE was purchased from Invitrogen Life Technologies and dissolved in DMSO as a 5 mM stock. After purification cells were washed three times in PBS and suspended at a concentration of 2 x10^7^ cells/ml in PBS. The CFSE stock was diluted to 10 μM in PBS and mixed with cells 1:1 (v/v), resulting in a final concentration of 5 μm CFSE. After 3 minutes, samples were vortexed and then continued incubating for an additional 2 minutes. After this time 1/10 of volume FCS was added for 1 minute followed by vortexing. The cells were then washed three times with complete medium and used in experiments.

### CD8^+^ T cell stimulation

For anti-CD3/CD28 stimulations, 48 well flat bottom plates were coated with 10 μg/ml of anti-CD3 and anti-CD28 antibodies or 20μg/ml control IgG in PBS overnight at 4°C. All antibodies were purchased from BD Pharmingen. After removal of PBS, purified T cells were added at 3x10^5^ cells per ml.

### Flow cytometry and FACS analysis

Samples were acquired on a CANTO II instrument (BD Biosciences) and data were analyzed using FloJo software (TreeStar).

### Detection of cell surface free thiols

Maleimide-Alexa Fluor 488, maleimide-Alexa Fluor 647, and maleimide-Pacific Blue were purchased from Invitrogen and dissolved in DMSO as 2.5mM stock. To assess CSFT, cells were incubated in 5μM maleimide-conjugate in 1% FCS in RPMI for 15 minutes on ice and then surface stained and fixed. In the pretreatment experiments cells were washed 3 times with PBS, followed by a 15 minute incubation with 5mM N-ethylmaleimide or dithiothreitol (DTT). Cells were then washed 3 times and incubated with fluorescently conjugated maleimide. 

### Surface and intracellular staining

In this study the following Abs were used: rat anti-mouse CD8α-PE, rat anti-mouse CD8 α -PerCP, rat anti-mouse CD8 α -APC, rat anti-mouse CD127-FITC, rat anti-mouse CD44-FITC, rat anti-mouse CD4-PE, rat anti-mouse CD90.1-FITC, rat anti-mouse KLRG1-PE, , rat anti-mouse CD69-PE, rat anti-mouse Granzyme B-PE, mouse anti-human Ki-67-PE and hamster anti-mouse PD-1. KLRG1 antibody was purchased from Abcam. CD127 and PD-1 antibodies were purchased from eBioscience. All other antibodies were purchased from BD Pharmingen. D^b^GP33-41, D^b^NP396-404, D^b^GP276-286 MHC class I tetramers were generated as previously described [12]. Surface staining was performed by incubation of Abs at a 1:100 dilution in FACS buffer for 30 min at 4°C. KLRG1 staining was performed at a 1:25 dilution. CD90.1 staining was performed at a 1:750 dilution. To measure intracellular Granzyme B levels, cells were treated with BD Biosciences Cytofix/Cytoperm kit according to the manufacturer’s instructions. To measure Ki-67 staining cells were stained with the BD FACS Lysing and Permeability Solutions kit according to the manufacturer’s instructions.

### Statistical analysis

Data were analyzed using a two-tailed student’s t test, and p< 0.05 was considered significant.

## Results

### Cell Surface Free Thiols Are Increased On Effector/Memory Compared To Naïve Phenotype CD8^+^ T Cells

**Figure 1:**
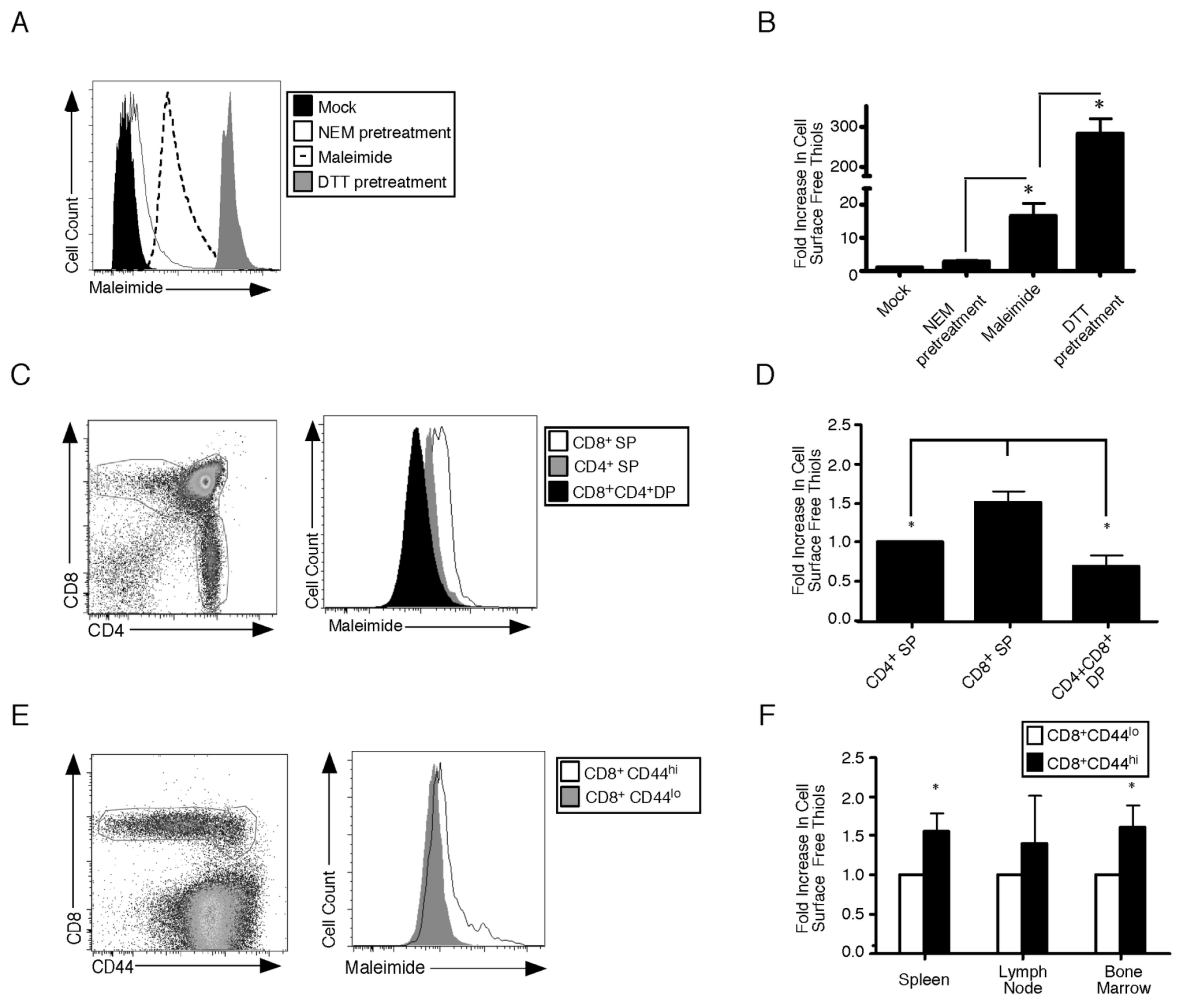
Cell Surface Free Thiols Are Increased On Memory Compared To Naïve Phenotype CD8^+^ T Cells. (A) Splenocytes from naive mice were pretreated with either media, 5mM N-ethylmaleimide (NEM) or 5mM dithiothretiol (DTT) and then incubated with media (mock) or 5μM maleimide-Alexa Fluor 488 and the fluorescence is plotted as a histogram. (B) The maleimide-Alexa Fluor 488 geometric mean for mock splenocytes was used as the baseline and the fold increase of the other populations was determined the average and standard deviation are plotted. (C) Thymocytes were isolated from naïve mice and then stained with anti-CD8α and anti-CD4 and maleimide-Alexa Fluor 488. The dot plot is gated on viable cells and the maleimide-Alexa Fluor 488 level for each population is plotted as a histogram (D) The maleimide-Alexa Fluor 488 geometric mean for CD4 SP T cells was used as the baseline and the fold increase of the other populations was determined the average and standard deviation are plotted. (E) Splenocytes were isolated from naïve mice and then stained with anti-CD8α and anti-CD44 and maleimide-Alexa Fluor 488. The dot plot is gated on viable cells and the maleimide-Alexa Fluor 488 level for each population is plotted as a histogram (F) The geometric mean for each population in the spleen, lymph node and bone marrow was determined and the average and standard deviation are plotted. Six mice were analyzed in two independent experiments. *, significant difference, P<0.05.

To understand how cell surface free thiols (CSFT) change with CD8^+^ T cell activation and differentiation we performed flow cytometry using fluorescently labeled conjugates of maleimide. This chemical probe specifically reacts with the thiol group found in cysteine to form a stable carbon sulfur bond and allows changes in surface protein oxidation to be monitored. To verify that staining was specific, splenocytes were pretreated with media, excess unlabeled maleimide or DTT and then incubated with maleimide conjugated with Alexa Fluor 488. Figure 1A & B show that pretreatment with maleimide decreases 88% of signal. Conversely, pretreatment with DTT resulted in a 15-fold increase cell surface free thiol staining. Taken together, these data demonstrate the probe is specifically measuring free thiols on the surface of cells. To understand how CSFT change during T cell development, thymocytes were isolated and stained with anti-CD8, anti-CD4 and fluorescently labeled maleimide (Figure 1C). CSFT were significantly higher on CD8 SP compared to CD4 SP or CD8CD4 DP T cells (Figure 1D). When splenocytes were interrogated CSFT levels were increased 1.5-fold in CD8^+^CD44^hi^ activated/memory cells compared to naïve CD8^+^CD44^low^ T cells (Figure 1E) and similar trends were observed in the lymph nodes and bone marrow (Figure 1F). Thus CSFT increase during T cell maturation and are higher on activated/memory compared to naïve phenotype CD8^+^ T cells. 

### Cell Surface Free Thiols Increase On Activated CD8+ T Cells Prior To The First Division

**Figure 2:**
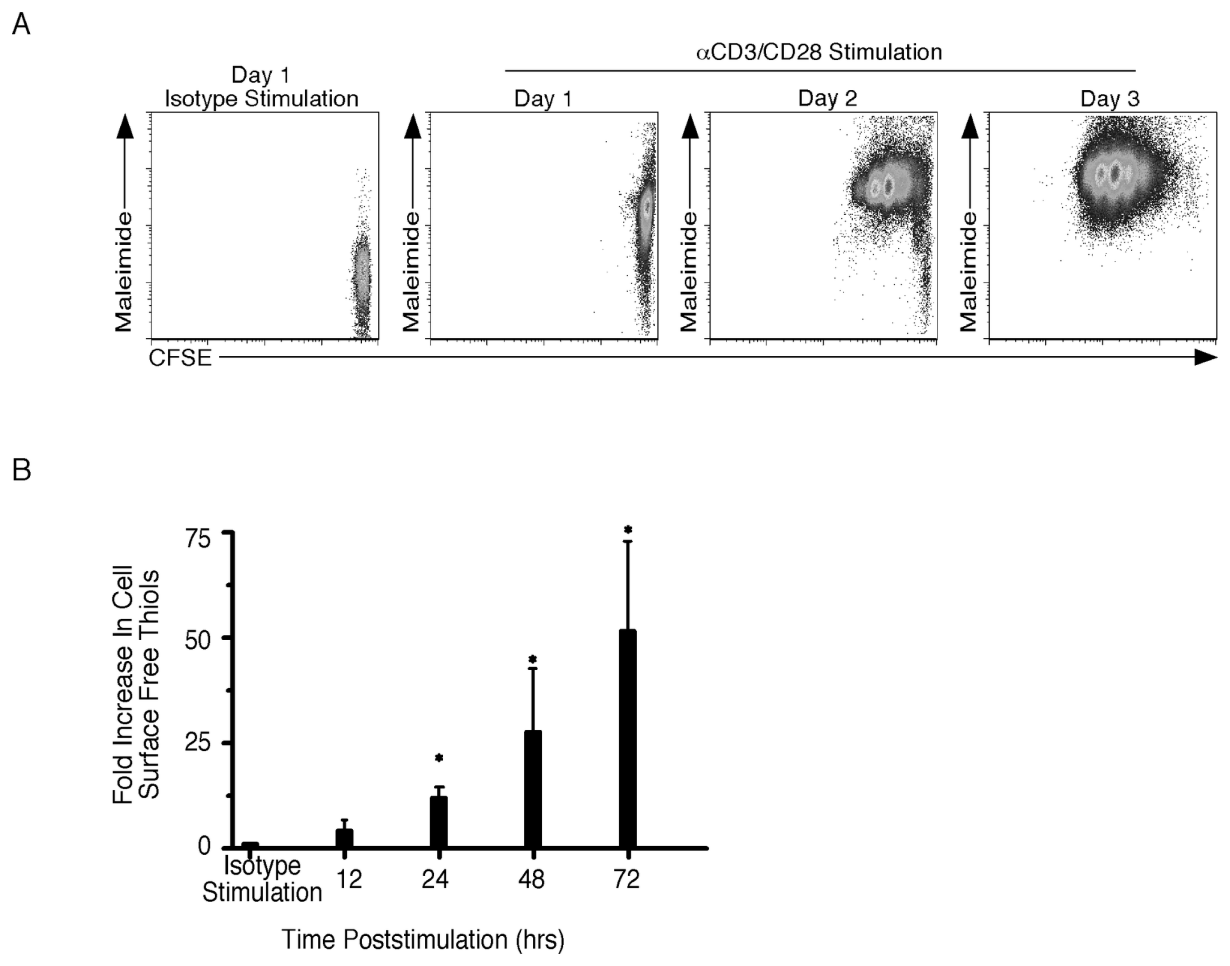
Cell Surface Free Thiols Increase On Activated CD8^+^ T Cells Prior To Division. Splenocytes were isolated from naïve mice and CD8^+^ T cells were purified and labeled with CFSE. Cells were then stimulated with either 10μg/mL isotype or anti-CD3 and anti-CD28. At the indicated timepoint, cells were harvested and incubated with maleimide-Alexa Fluor 647. Proliferation (A) was assessed by loss of CFSE fluorescence after activation. (B) The fold increase in cell surface free thiols relative to those found on isotype stimulated cells was determined and the average and standard deviation are plotted. Six mice were analyzed in two independent experiments. *, significant difference, P<0.05.

To understand how CSFT change on CD8^+^ T cells during activation, purified T cells were labeled with CFSE and CSFT levels were assessed as cells began to divide. Figure 2A shows that after 24 hours, isotype stimulated CD8^+^ T cells had low levels of CSFT whereas those on anti-CD3/CD28 stimulated cells had increased. Importantly, this increase was independent of CFSE dilution demonstrating that it did not require division. From day 1 to 3 cells underwent multiple divisions and the CSFT levels increased further. When the results were quantitated from multiple experiments ~3-fold increase in CSFT could be observed at 12 hours compared to isotype stimulated cells (Figure 2B). By 24 hours CSFT levels increased 10-fold compared to isotype treated cells and this continued to rise at 48 and 72 hours. In contrast incubation with IFNα or IL-12 did not raise CSFT above the levels detected on isotype treated cells (data not shown). Thus CSFT levels initially increase prior to division and continue to rise as cells proliferate.

### Cell Surface Free Thiols Are Increased On Effector Compared To Memory CD8^+^ T Cells During Acute Viral Infection

**Figure 3:**
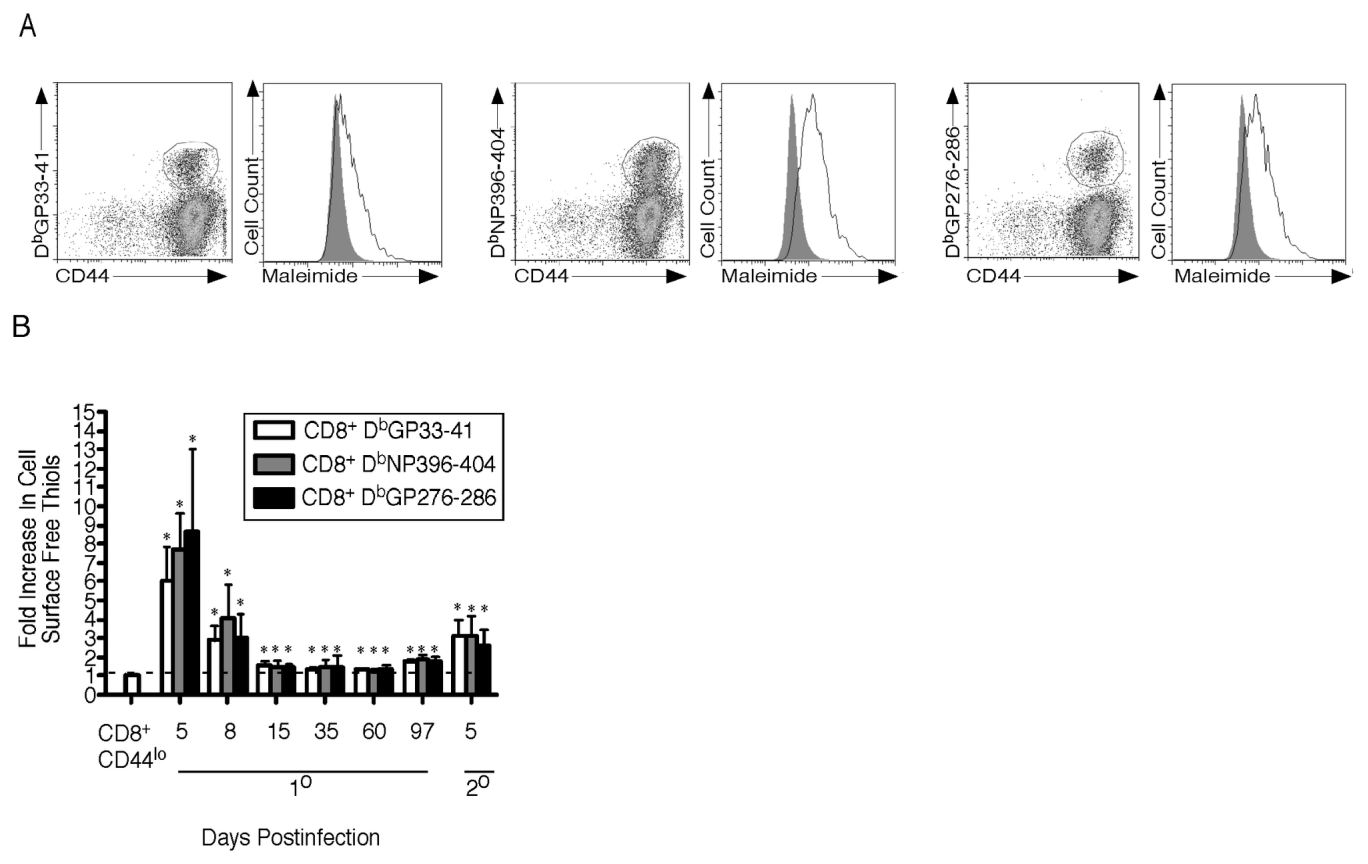
Cell Surface Free Thiols Are Higher On Effector Compared To Memory CD8^+^T Cells During Acute Infection. (A) Mice were infected with LCMV-Armstrong and on day 8 postinfection splenocytes were harvested and stained with anti-CD8α, anti-CD44, the indicated MHC Class I tetramer and maleimide-Alexa Fluor 488. The maleimide-Alexa Fluor 488 levels of the gated populations are plotted in the histogram format with the shaded area indicating the staining of CD8^+^CD44^lo^ T cells from naïve mice. (B) Mice were examined at multiple times following LCMV- Armstrong (1°) infection or Clone 13 rechallenge (2°) and the fold increase in cell surface free thiols relative to naïve phenotype cells from uninfected mice was determined and the average and standard deviation are plotted. Six mice were analyzed in two independent experiments. *, significant difference, P<0.05.

To understand how CSFT levels change in vivo as CD8^+^ T cells become activated and differentiate we infected mice with LCMV-Armstrong. This virus induces an acute infection that will be cleared in 8 to 9 days and will generate lifelong CD8^+^ T cell-mediated immunity. Using MHC Class I tetramers to three D^b^-restricted LCMV epitopes (GP33-41, NP396-404, and GP276-286) we determined that CSFT were increased ~4-fold on antigen-specific CD8^+^ T cells at the peak of the effector response compared to naïve phenotype (CD8^+^CD44^low^) T cells from uninfected mice (Figure 3A). When an extended timecourse was examined the greatest levels of CSFT (7-9 fold increase) were observed at day 5 postinfection when high levels of virus are present (Figure 3B). From day 5 to 8 postinfection antigen-specific CD8^+^ T cells increase 10-fold, but the levels of CSFT decline on a per cell basis. After viral clearance, the levels of CSFT decline further and by the time the memory phase was reached levels were elevated slightly compared to naïve cells. When LCMV-Armstrong infected mice were rechallenged with LCMV-Clone 13 a vigorous secondary expansion was induced that peaked 5 days postinfection [11]. The CSFT levels on secondary effector CD8^+^ T cells increase ~4-fold compared to those found on naïve T cells reaching a level comparable to those on primary effector cells at the peak of the response on day 8 (Figure 3B). Thus CSFT are elevated on both primary and secondary effector compared to naïve and memory CD8^+^ T cells.

### Cell Surface Free Thiols Are Highest On Antigen-Specific CD8^+^ T Cells In Lymphoid Organs

**Figure 4:**
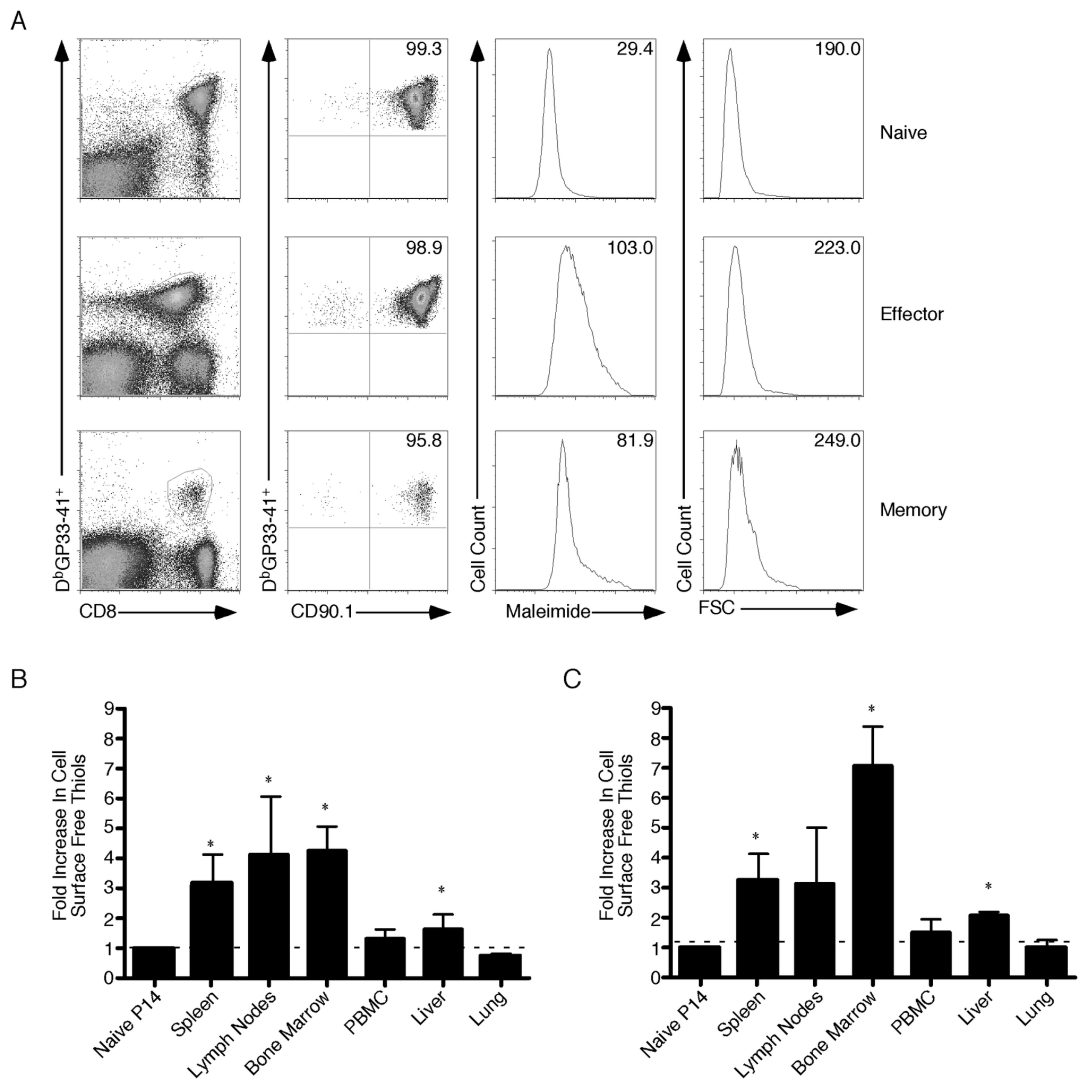
Cell Surface Free Thiols Are Highest On Antigen-Specific Cells In Lymphoid Tissues. (A) A total of 10^4^ naïve P14 cells were transferred into naïve C57BL/6 hosts. Four hours later mice were infected with LCMV-Armstrong and harvested either 8 (Effector) or 79 (Memory) days later. Splenocytes were incubated with maleimide-Alexa Fluor 488,anti-CD8α, anti-CD90.1 and D^b^GP33-41 MHC Class I tetramer. The maleimide-Alexa Fluor 488 and forward scatter levels of the gated populations are plotted in the histogram format with the geometric mean indicated by the value in the upper right hand corner of the plot. The fold increase in cell surface free thiols compared to naïve P14 cells during either the effector (B) or memory (C) phases was determined for P14 cells in the indicated tissues. The average and standard deviation are plotted. Six mice were analyzed in two independent experiments. *, significant difference, P<0.05 between naïve P14 and cells from the indicated tissues.

To understand how CSFT change on CD8^+^ T cells in various tissues during infection we utilized P14 TCR transgenic mice. These mice express a TCR that recognizes the D^b^-restricted epitope GP33-41 of LCMV. These cells also allow direct measurement of CSFT changes on the same cell as it differentiates from naïve to effector to memory. Figure 4A shows that following adoptive transfer of a small number of Thy1.1^+^ P14 cells into congenic C57BL/6 hosts a massive expansion was induced and the D^b^GP33-41 specific response was almost completely transgenic. When CSFT were measured, naïve P14 cells had a geometric mean of 29.4 that increased to 103 by day 8 postinfection (effector) and declined to 81.9 when the memory phase was reached. Importantly, there was minimal difference in the forward scatter of the various populations demonstrating the changes observed in CSFT level were not solely due to cell size. During LCMV infection antigen-specific CD8^+^ T cells expanded in multiple tissues. On day 8 (Figure 4B) and 79 (Figure 4C) postinfection cells were harvested from various organs and the CSFT levels were measured. During both the effector and memory phases CSFT levels were the highest in lymphoid organs such as the spleen, lymph nodes and bone marrow. Indeed the CSFT levels in the lymph nodes and spleen either decreased (lymph nodes) or remained constant (spleen), but in the bone marrow staining increased between effector and memory CD8^+^ T cells. When nonlymphoid tissues were examined, CSFT levels were always less than lymphoid tissues and were lower at both timepoints in the lung. Thus CSFT are highest on antigen-specific CD8^+^ T cells in lymphoid tissues.

**Figure 5:**
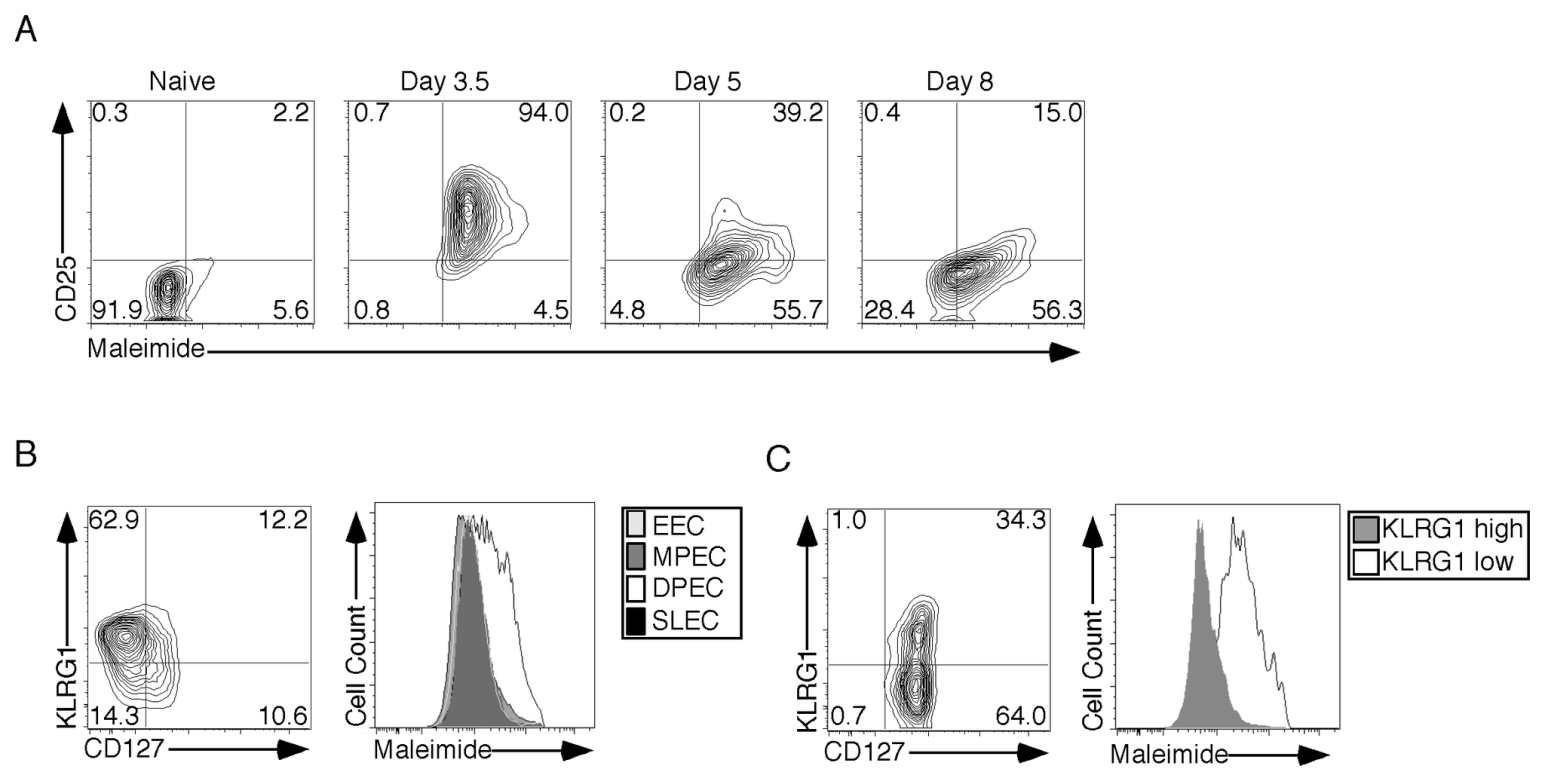
Double Positive Effector Cells Have the Largest Increase In Cell Surface Free Thiols. To assess cell surface free thiol expression during the early differentiation of CD8^+^ T cells a total of 10^5^ naïve P14 cells were transferred into naïve C57BL/6 hosts. Four hours later mice were infected with LCMV-Armstrong and harvested at the indicated timepoint. (A) Splenocytes were incubated with maleimide-Pacific Blue, anti-CD8α, anti-CD90.1 and anti-CD25. Dot plots are gated on CD8^+^CD90.1^+^ T cells and the value in the upper right hand corner of the plot indicates the percent of cells in that quadrant. (B) To determine the expression of cell surface free thiols during later timepoints in the differentiation process a total of 10^4^ naïve P14 cells were transferred into naïve C57BL/6 hosts. Four hours later mice were infected with LCMV-Armstrong and harvested at the indicated timepoint. Splenocytes were incubated with maleimide-Alexa Fluor 488, anti-CD8α, anti-CD90.1, anti-CD127 and anti-KLRG1. The expression of cell surface free thiols was examined in the effector (B) or memory (C) phases using P14 cells. Dot plots are gates on CD8^+^CD90.1^+^ T cells and the value in the upper right hand corner of the plot indicates the percent of cells in that quadrant. The maleimide-Pacific Blue level of the gated population is plotted in the histogram format.

 To better understand the relationship between CSFT and T cell differentiation we adoptively transferred P14 cells and examined the early differentiation of effector CD8^+^ T cells. Prior studies by Kalia and colleagues have demonstrated that prolonged IL-2 receptor alpha chain expression (CD25) is correlated with terminal differentiation into CD127^low^KLRG1^high^ short lived effector cells (SLEC) [13]. In Figure 5A effector cells from days 3.5 to 8 postinfection were examined for the expression of CD25 and CSFT. On day 3.5 postinfection ~94% of effector cells expressed a broad range of CD25. Interestingly, cells with an intermediate level of CD25 had the highest level of CSFT. As the infection progressed CD25 levels declined on day 5 and by day 8 postinfection almost all of the cells had CD25 expression similar to naïve T cells. At the later timepoints P14 cells that possessed increased expression of CD25 had the highest level of CSFT. Thus early during effector differentiation cells with intermediate levels of CD25 expression possessed the highest levels of CSFT, but as differentiation proceeded cells that maintained their CD25 expression also had high CSFT. 

 Effector CD8 T cell development culminates with differentiation into four subsets: CD127^low^KLRG1^low^ early effector cells (EEC), CD127^low^KLRG1^high^ short lived effector cells (SLEC), CD127^high^KLRG1^high^ double positive effector cells (DPEC) and CD127^high^KLRG1^low^ memory precursor cells (MPEC) [14]. Most of the SLEC will undergo apoptosis while the MPEC, and DPEC to a lesser extent, will give rise to long-lived memory cells. By day 8 postinfection most of the P14 cells had differentiated into SLEC (62.9%, Figure 5B), while the remaining cells were split amongst the other populations. When CSFT were measured, DPEC had the highest level while the other three populations expressed similar amounts. In the memory phase (Figure 5C) a mixture of KLRG1^high^ and low cells remained 79 days postinfection. Again CSFT were the highest on CD127^high^KLRG1^high^ T cells. Thus CSFT are increased to the greatest extent on CD127^high^KLRG1^high^ CD8^+^ T cells during both the effector and memory phases.

### Prolonged Elevation Of Cell Surface Free Thiols During Chronic Viral Infection

**Figure 6:**
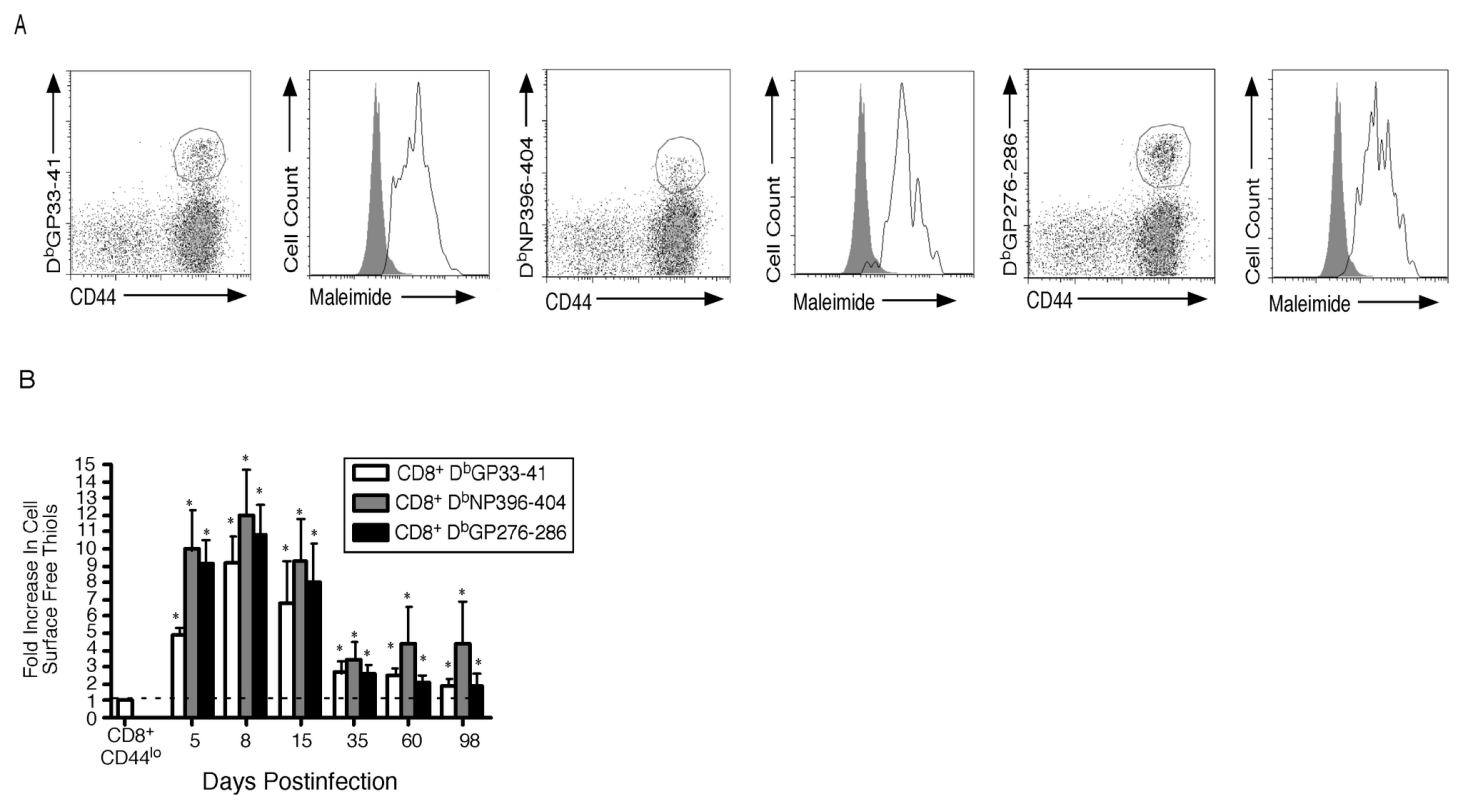
Cell Surface Free Thiols Remain Elevated On Antigen-Specific CD8^+^ T Cells For Extended Periods During Chronic Viral Infection. (A) Mice were infected with LCMV-Clone 13 and on day 8 postinfection, splenocytes were harvested and stained with anti-CD8α, anti-CD44, the indicated MHC Class I tetramer and maleimide-Alexa 488. The maleimide-Alexa Fluor 488 level of the gated populations is plotted in the histogram format with the shaded area indicating the staining of CD8^+^CD44^lo^ T cells from naïve mice. (B) Mice were examined at multiple times following LCMV-Clone 13 infection and the fold increase in cell surface free thiols relative to naïve phenotype cells from uninfected mice was determined and the average and standard deviation are plotted. Six mice were analyzed in two independent experiments. *, significant difference, P<0.05.

Because CSFT were elevated during the effector phase of an acute antiviral immune response we wanted to determine levels under conditions where antigen persisted. To address this question we infected mice with LCMV-Clone 13 that induces a chronic viral infection where antigen persists in tissues for extended periods [15]. At the peak of effector response on day 8 postinfection, splenocytes were harvested and CSFT levels were measured on antigen-specific CD8^+^ T cells using LCMV-specific tetramers. Figure 6A shows that CSFT were increased 9 to 12-fold compared to naïve phenotype cells from uninfected mice. When early timepoints were examined (Figure 6B), similar to acute infection CSFT were increased ~10-fold on day 5 postinfection. From day 5 to 8 postinfection CSFT levels remained constant and this was maintained though 15 days. By day 35 postinfection, when viral load is lowered in the spleen, CSFT levels had decreased although these were still higher than those found during acute infection. Thus CSFT remain elevated on antigen-specific CD8^+^ T cells for extended periods during chronic infection.

### Cell Surface Free Thiol Expression Identifies Effector CD8^+^ T Cells Undergoing Recent TCR Stimulation

**Figure 7:**
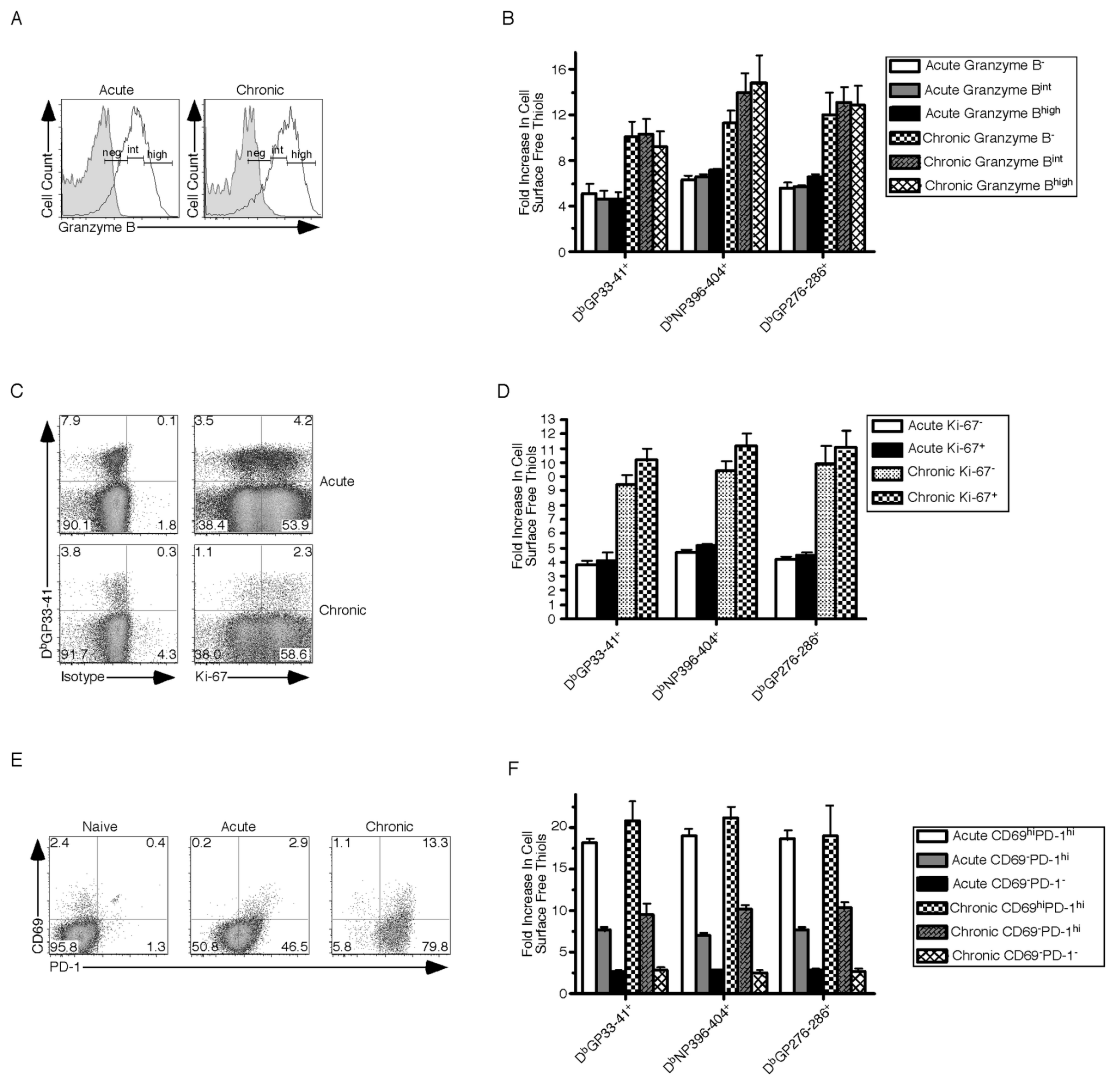
Cell Surface Free Thiols Are Elevated On Antigen-Specific Cells Undergoing Recent TCR Stimulation. Naïve mice were infected with either LCMV-Armstrong (Acute) or LCMV-Clone 13 (Chronic) and harvested 8 days postinfection. To determine if cell surface free thiol levels correlated with expression of granzyme B (A), splenocytes were stained with anti-CD8α, D^b^GP33-41, isotype control or anti-Granzyme B and maleimide-Alexa Fluor 488. The histogram is gated on CD8^+^D^b^GP33-41^+^ T cells and the shaded area indicates isotype staining. For each population the Granzyme B expression was divided into negative (neg), intermediate (int) , and high (high) levels of staining. (B) The cell surface free thiol levels compared to those on CD8^+^CD44^lo^Granzyme B^-^ T cells from uninfected mice were determined for each antigen-specific population and the average and standard deviation are plotted. To determine whether cell surface free thiols were elevated on antigen specific CD8^+^ T cells that were actively proliferating (C) splenocytes were stained with anti-CD8α, D^b^GP33-41, isotype control or anti-Ki-67, and maleimide-Alexa Fluor 488 . Dot plots are gated on total CD8^+^ T cells and the values in each corner indicate the percent of cells that are in that quadrant. (D) The cell surface free thiol levels compared to those on CD8^+^CD44^lo^Ki-67^-^ T cells from uninfected mice were determined for each antigen-specific population and the average and standard deviation are plotted. (E) To determine recent TCR stimulation splenocytes were stained with anti-CD8α, anti-CD69, anti-PD-1, D^b^GP33-41 and maleimide-Pacific Blue. The dot plots are gated on either CD8^+^CD44^lo^ (naïve) or CD8^+^D^b^GP33-41^+^ (infected) T cells. The value in each corner indicates the percent of cells in that quadrant. (F) The cell surface free thiol levels compared to those on CD8^+^PD-1^-^CD69^-^ T cells from uninfected mice were determined for each antigen-specific population and the average and standard deviation are plotted. Six mice were analyzed in two independent experiments. *, significant difference between acute and chronic infection, P<0.05.

Prior studies have suggested cells with increased CSFT have a greater functional capacity [16], yet our results document that CSFT are higher on antigen-specific CD8^+^ effector T cells from chronic LCMV infection compared to memory cells generated following acute LCMV infection. This distinction is critical because during chronic infection virus-specific CD8^+^ T cells undergo exhaustion decreasing cytokine production and cytolysis, whereas polyfunctional memory cells are rapidly able to produce cytokines and kill infected cells [15]. Because agents such as monensin, which block transit through the ER, decrease CSFT, assessing function by intracellular cytokine staining is problematic. To measure functional capacity in the absence of peptide restimulation we performed intracellular staining for Granzyme B. Splenocytes were harvested from day 8 LCMV-Armstrong (acute infection) or Clone 13 (chronic infection) and surface stained with anti-CD8α, anti-CD44, D^b^GP33-41 and maleimide-Pacific Blue. Figure 7A shows that during both infections a range of Granzyme B staining was observed in antigen-specific CD8^+^ T cells. CSFT were determined for LCMV-specific CD8^+^ T cells that had negative, intermediate or high levels of Granzyme B staining. Similar to our prior results, CSFT levels were higher across all three epitopes in chronic compared to acute infection but there was no correlation with granzyme B expression (Figure 7B). In addition to decreased function antigen-specific CD8^+^ T cells from acute versus chronic viral infection undergo differential proliferation and TCR stimulation. To identify proliferating cells MHC Class I tetramer^+^ cells were costained with maleimide-Pacific Blue and anti-Ki-67. In both acute and chronically infected mice most of the D^b^GP33-41 specific CD8^+^ T cells were undergoing division although a greater percentage in chronic infection (Figure 7C) were dividing. When multiple epitopes were examined (Figure 7D), CSFT were again increased on effector cells from chronic compared to acute infection, but no differences were observed between Ki-67 positive and negative cells. Since antigen persists for extended periods in chronic compared to acute infection, we determined whether CSFT level identified effector CD8^+^ T cells undergoing recent TCR stimulation. Upon antigen stimulation CD8^+^ T cells upregulate surface levels of CD69 and the inhibitory receptor programmed death-1 (PD-1) [17]. By day 8 of acute infection only a small proportion (~2.9%) of CD8^+^D^b^GP33-41^+^ T cells are positive for both markers while 46.5% express increased PD-1 (Figure 7E). This contrasts with chronic infection where by day 8 postinfection 13.3% of D^b^GP33-41 specific CD8^+^ T cells express high levels of both markers and 79.8% express elevated PD-1. When CSFT were determined CD69^high^PD-1^high^ cells, regardless of the infection they were isolated from or antigen-specificity, had the highest staining (Figure 7F). The next highest levels were observed on CD69^-^PD-1^high^ cells while the CSFT on CD69^-^PD-1^-^ cells were the lowest of any of the populations. Taken together these results demonstrate that the level of CSFT on effector CD8^+^ T cells indicates recent TCR stimulation not functional capacity or proliferative status.

## Discussion

In this study we examined the expression of CSFT during CD8^+^ T cell development, activation, proliferation and differentiation during viral infection. Here we report seven novel observations. First, CSFT are increased on developing CD8 SP compared to CD4 SP or CD8CD4 DP T cells in the thymus. Second, after emigration to the periphery CSFT are higher on activated/memory compared to naïve phenotype CD8^+^ T cells. Third, during activation CSFT increase on CD8^+^ T cells prior to division, and become further elevated with proliferation. Fourth, during acute viral infection CSFT become elevated on antigen-specific effector CD8^+^ T cells and decline as they differentiate into memory cells. Fifth, CSFT are highest on antigen-specific CD8^+^ T cells from lymphoid tissues during infection. Sixth, during chronic viral infection, CSFT are elevated on antigen-specific CD8^+^ T cells for extended periods. Seventh, during infection the level of CSFT identifies effector cells undergoing recent TCR stimulation not active cycling or expression of cytolytic molecules.

 What are the implications of our observed changes in CSFT for early activation? It is well established that naïve CD8^+^ T cells require three signals for optimal activation and differentiation: (i) TCR recognition of cognate peptide MHC complexes, (ii) interaction with costimulatory molecules and (iii) programming by inflammatory cytokines [18]. Recently evidence has emerged that production of ROI is another critical event for T cell activation. Although levels are low in naïve CD8^+^ T cells, within 15 minutes of cognate peptide stimulation ROI increase 100% [3]. This increase is essential for activation as antioxidant treatment, which lowers ROI, decreases T cell activation [4]. Since H_2_O_2_ can readily diffuse across membranes mechanisms must exist to prevent inappropriate surface receptor oxidation and allow sustained TCR signaling. Here we show that within hours of naïve CD8^+^ T cell activation in vitro the total level of CSFT increases prior to division. As cells divide CSFT levels increase further. Exposure of naïve CD8^+^ T cells to costimulation or inflammatory cytokines in the absence of TCR stimulation had minimal effect (data not shown). Taken together these data suggest ROI and CSFT rise in a coordinated fashion in response to TCR stimulation to prevent overoxidation of the cell surface. The source of increased CSFT is unclear. Formally the increase observed in our and other studies could be due to new protein synthesis and export to the surface or reduction of disulfide bonds in surface proteins. Evidence for localization of new proteins to the surface is provided by studies using monensin which decreased CSFT on human PBMCs [9]. Alternatively treating cells with N-Acetyl-cysteine or an analog that cannot be used in intracellular glutathione synthesis, N-acetyl-D-cysteine raises CSFT, supporting the idea that changes in the extracellular redox state can modulate CSFT [19]. Identifying cell surface proteins with free thiols has proven elusive. To date only a small number of candidates that are exclusively surface proteins have been identified. Using a proteomics based approach Laragione and colleagues demonstrated that CSFT in VLA-4 increased with NAC treatment and that this modulated the interaction with fibronectin in an adhesion assay [19]. In addition to VLA-4 other immunologically relevant protein-protein interactions may be sensitive to CSFT status. Prior studies by Kanner and colleagues have shown that the interaction between CD4 and lck is blocked by NEM suggesting free thiols are critical for this interaction [20]. In addition to TCR-MHC-peptide interactions, cytokine signaling is critical for T cell activation. Prior studies by Smith and colleagues demonstrated agents that bind to CSFT decrease responsiveness to IL-2 [8]. Although there is no evidence to date that the interaction between costimulatory molecules and their receptors on CD8^+^ T cells is regulated by oxidation, it is tantalizing to speculate that this may be another extracellular redox mechanism to fine tune T cell responses similar to the modulation of cystine and cysteine levels by dendritic cells [21]. 

Taken together our results suggest that CSFT are dynamically regulated in vivo during CD8^+^ T cell activation, proliferation and differentiation. During development we observed that CD8^+^ SP T cells had increased CSFT compared to those on CD4 SP or CD4^+^CD8^+^ DP T cells. This confirms and extends prior studies by Lawrence [9] and Sahaf et. al that documented CSFT levels were higher on CD8^+^ compared to CD4^+^ T cells isolated from patient’s PBMCs [22]. In vivo we observed that early during infection (3.5-5 days), when antigen and inflammatory cytokines would both be increased, CSFT were elevated 10-fold or greater on antigen-specific CD8^+^ T cells. In our prior studies we documented that superoxide levels were increased in P14 cells 5 days postinfection compared to naïve cells [23]. This again supports a role for CSFT in preventing overoxidation of the cell surface. In vivo, as the infection is brought under control from day 5 to 8, superoxide and CSFT decline on antigen-specific CD8^+^ T cells. Importantly the levels of CSFT were not equivalent amongst all effector cells. We observed that EEC, SLEC and MPEC all had similar levels of CSFT, while the highest levels were found on DPEC. This subset of effector cells expresses high levels of CD127 and KLRG1 and a portion of these cells will survive 181 days into the memory phase [14]. Although there are probably multiple proteins with CSFT, murine KLRG1 has 10 cysteines in its extracellular domain and these are used to form multiple disulfide bonds both within the molecule and to facilitate dimerization for interaction with E-cadherin [24,25]. This raises the question whether the disulfide bond formation is altered in DPEC compared to SLEC and how this would affect KLRG1’s ability to negatively regulate TCR signaling. In memory CD8^+^ T cells superoxide levels are lower than those found in naïve CD8^+^ T cells, but CSFT are modestly elevated. Interestingly, memory cells from later timepoints (days 79 or 97) have increased CSFT compared to those from day 35 postinfection. This increase in CSFT may represent another aspect in memory differentiation similar to the conversion of CD62L^low^ effector to CD62L^high^ central memory cells that occurs following infection [26]. In support of this idea human CD8^+^ T cells treated with IL-15 for a week during in vitro culture upregulate CSFT [27]. Our results are also the first to measure CSFT levels on antigen-specific effector CD8^+^ T cells during acute and chronic viral infection. We have clearly shown that CSFT remain elevated for prolonged periods on antigen-specific CD8^+^ T cells during chronic infection and are not a marker of cytolytic capacity or proliferative status but rather indicate recent TCR stimulation. In support of this idea, Sahaf and colleagues demonstrated that CSFT were higher on CD4^+^ T cells from HIV patients compared to healthy controls [22]. Although the antigen specificity of these populations was not determined, in HIV patients there should be more activated CD4^+^ T cells with increased CSFT. But, our studies contrast with those by Gelderman and colleagues [16] who found that T cell surface redox levels determine T cell reactivity and arthritis susceptibility in an autoimmune model. In their study increased CSFT were associated with increased IL-2 production, division and ability to cause autoimmune disease following adoptive transfer. Since they examined self-reactive CD4^+^ T cells it is consistent with our studies that cells undergoing self-stimulation in vivo would be CD69^high^ and PD-1^high^ and possess increased CSFT. But our data clearly demonstrate that CSFT do not correlate with the cytolytic capacity or proliferative status of effector CD8^+^ T cells. Taken together they argue the role of CSFT in T cell function is complex and may be differentially regulated between CD4^+^ and CD8^+^ T cells during viral infection and autoimmunity.

In conclusion we have shown that CSFT increase during the activation, proliferation and differentiation of CD8^+^ T cells. CSFT levels are highest on antigen-specific CD8^+^ T cells in lymphoid organs and remain elevated during chronic infection. Importantly, increased CSFT on antigen-specific effector CD8^+^ T cells identifies cells that have undergone TCR stimulation not the expression of cytolytic molecules or proliferative status. The demonstration that increased CSFT mark recently stimulated effector T cells provides another marker to understand T cell differentiation during infection and to develop therapies to remove these cells during autoimmunity, graft versus host disease and transplantation.
